# The Effect of Heavy Metals on Conjugation Efficiency of an F-Plasmid in *Escherichia coli*

**DOI:** 10.3390/antibiotics11081123

**Published:** 2022-08-19

**Authors:** Martin Palm, Alfred Fransson, Julia Hultén, Karolina Búcaro Stenman, Amina Allouche, Oscar E. Chiang, Mirthe L. Constandse, Karlijn J. van Dijk, Suheda Icli, Bela Klimesova, Emma Korhonen, Gema Martínez-Crespo, Dominik Meggers, Margarita Naydenova, Maria An. Polychronopoulou, Dominik B. Schuntermann, Havva Unal, Agnieszka Wasylkowska, Anne Farewell

**Affiliations:** 1 Department of Chemistry and Molecular Biology, University of Gothenburg, 40530 Gothenburg, Sweden; 2 Centre for Antibiotic Resistance Research (CARe), University of Gothenburg, 40530, Gothenburg, Sweden

**Keywords:** bacterial conjugation, *Escherichia coli*, heavy metals, plasmids, antibiotic resistance, horizontal gene transfer

## Abstract

Conjugation, the process by which conjugative plasmids are transferred between bacteria, is regarded as a major contributor to the spread of antibiotic resistance, in both environmental and clinical settings. Heavy metals are known to co-select for antibiotic resistance, but the impact of the presence of these metals on conjugation itself is not clear. Here, we systematically investigate the impact that five heavy metals (arsenic, cadmium, copper, manganese, and zinc) have on the transfer of an IncF conjugative plasmid in *Escherichia coli*. Our results show that two of the metals, cadmium and manganese, have no significant impact, while arsenic and zinc both reduce conjugation efficiency by approximately 2-fold. Copper showed the largest impact, with an almost 100-fold decrease in conjugation efficiency. This was not mediated by any change in transcription from the major Py promoter responsible for transcription of the conjugation machinery genes. Further, we show that in order to have this severe impact on the transfer of the plasmid, copper sulfate needs to be present during the mating process, and we suggest explanations for this.

## 1. Introduction

Antibiotic resistance (ABR) is a growing crisis, with resistance spreading worldwide and new resistant strains emerging rapidly. In 2019, there were an estimated 4.95 million deaths associated with bacterial antimicrobial resistance, with 1.27 million deaths directly attributed to bacterial ABR [1]. One of the worst pathogens, in terms of deaths due to resistance, is *Escherichia coli*, with more than 250,000 deaths estimated in 2019 [1]. *Enterobacteriaceae* (including *E. coli*) carrying extended spectrum beta-lactamases (ESBL) are resistant to third-generation cephalosporins, and, together with carbapenem resistant *Enterobacteriaceae*, they have been classified as a critical priority by the WHO [2].

A major driving force behind the spread of antibiotic resistance is the horizontal transfer of resistance between bacteria, particularly through conjugation [3]. During conjugation, a donor cell uses a pilus to transfer a conjugative plasmid to a recipient cell, thus passing on the genes carried by the plasmid. Most clinically relevant resistance genes have been found on conjugative plasmids, including those with resistance to the last-resort antibiotic, colistin [4]. Bacterial conjugation is a complex and diverse process that is defined on a plasmid-to-plasmid basis. Traditionally, plasmids are typically grouped into incompatibility (Inc) groups based on their inability to be stably propagated together with other plasmids that share similar systems for replication and partitioning. In *Enterobacteriaceae* there are 28 Inc groups and at least four (IncF, I, A/C, and H) are notoriously responsible for ABR [5]. Conjugative plasmids have also been grouped based on the machinery necessary for conjugation: the mobility (MOB) type, where plasmids are grouped based on the amino acid sequence of the plasmid-encoded relaxase [6]; and the mating pair formation (MPF) type, which is determined by the protein homology of the type IV secretion system (T4SS) and auxiliary systems [7]. There is a wide array of bacterial plasmid sequences and proteins, and in addition, some plasmid families have been found to require chromosomally encoded factors [8,9,10,11], making the host strain an important factor in the plasmid’s ability to transfer. 

Inhibiting conjugation has been suggested as a way to decrease the rate at which antibiotic resistance spreads (reviewed in [12]), and several compounds have been shown to affect conjugation. For example, studies have shown that unsaturated fatty acids, alkynoic fatty acid derivatives, synthetic fatty acids, and tanzawaic acids have an inhibitory effect on conjugation [13,14,15,16]. On the other hand, studies have also shown that sub-inhibitory concentrations of antibiotics such as fluoroquinolones or tetracyclines can actually promote conjugation [17,18]. Furthermore, heavy metals have been suggested to both inhibit and promote conjugation, as described below.

Heavy metals occur naturally as part of the Earth’s crust. They can contaminate soil and water through natural processes, such as metal corrosion, leaching of heavy metals from soil to water, sediment resuspension, and evaporation from water sources to soil. However, most environmental contamination occurs through human activities, such as mining, industrial production, and the use of heavy metal-containing compounds in agriculture; for example, in animal feed and pesticides [19,20]. Heavy metals have been used as antimicrobials and disinfectants for thousands of years [21]. In bacteria, one major cause of heavy metal-mediated toxicity is the generation of reactive oxygen species (ROS) that can damage proteins and DNA or destabilize the integrity of lipid membranes by lipid peroxidation [22]. Additionally, heavy metals can interfere with the function of enzymes, either by binding covalently to functional groups in the active site, or by competing with and displacing other essential ions in the active site, thus blocking their function [22,23,24]. However, while most heavy metals are associated with toxicity, many of them are also essential in trace amounts for many biological and biochemical processes, where they make up important components of key enzymes [25]. Maintaining heavy metal homeostasis, where intracellular levels are kept high enough to maintain enzyme function and low enough to not cause toxicity, is thus essential for the cell’s survival [26]. 

The effect of heavy metals on conjugation and antibiotic resistance is complex [27]. It has been known for decades that bacteria exhibit metal and antibiotic resistance co-selection. Most commonly, this is due to the presence of genetically linked antibiotic and metal resistance genes [28]. For example, Sandegren et al. (2012), isolated a plasmid from *Klebsiella pneumoniae* that encodes resistance to six different antibiotics as well as to heavy metal ions (silver, copper, and arsenic). Thus, selection for any of these resistances will be for the plasmid carrying multiple resistances [29]. Co-selection of metal and antibiotic resistance can also occur through cross-resistance (with one determinant responsible for both resistances, e.g., efflux pump), or through co-regulation (reviewed in [27,28]).

In addition to co-selection, heavy metals have been reported to affect conjugation rates. The heavy metals arsenic (As), cadmium (Cd), copper (Cu), and zinc (Zn) have previously been reported to impact the ability of *Enterobacteriaceae* to transfer a conjugative plasmid through conjugation [30,31,32,33,34,35]. However, some of the data are contradictory (e.g., [30,34,36]), possibly because these studies were performed in such different ways. Specifically, it is difficult to generalize the results from one type of plasmid in one or a few species to most plasmids/species. In addition, it can be difficult to separate co-selection of ABR in the presence of heavy metals from conjugation itself. Thus, each type of plasmid needs to be tested in a systematic way. In this work we investigate how five heavy metals affect the conjugation efficiency of an IncF plasmid in *Escherichia coli*. 

## 2. Results

### 2.1. Effect of Heavy Metals on Growth

To determine the optimal concentration of heavy metals to use in our conjugation assay, i.e., one that has a slight observable effect on cell growth but does not completely arrest growth, we performed an experiment where we added various concentrations of the chemicals to log phase HA14 (donor) cells. The chemicals and concentrations tested are listed in Table 1. These concentrations were chosen based on a literature search to find MIC values determined by other studies [30,35,37,38,39,40,41,42,43,44,45,46]. Based on these results (Figure S1), we chose the following concentrations for the subsequent experiments: 0.2 mM NaAsO_2_, 2.0 mM CuSO_4_, 0.2 mM CdSO_4_, 5.0 mM MnCl_2_, and 0.37 mM ZnCl_2_. 

### 2.2. Conjugation of the F-Plasmid Is Inhibited by Copper Sulfate

To investigate the effect that the various chemicals have on conjugation efficiency, we performed a liquid conjugation assay where log phase donor and recipient cells were mixed and allowed to mate in the presence of the heavy metal for 30 min at 37 °C. The mixtures were then diluted and spotted onto plates selecting for transconjugants (Chl, Tet) and donors (Tet, Kan), after which the conjugation efficiency was defined as the ratio of transconjugants formed per donor cell. A control mating (without metals) was carried out each day to normalize day-to-day variation. These control matings showed an average of 45% conjugation efficiency (20% standard deviation).

**Figure 1:**
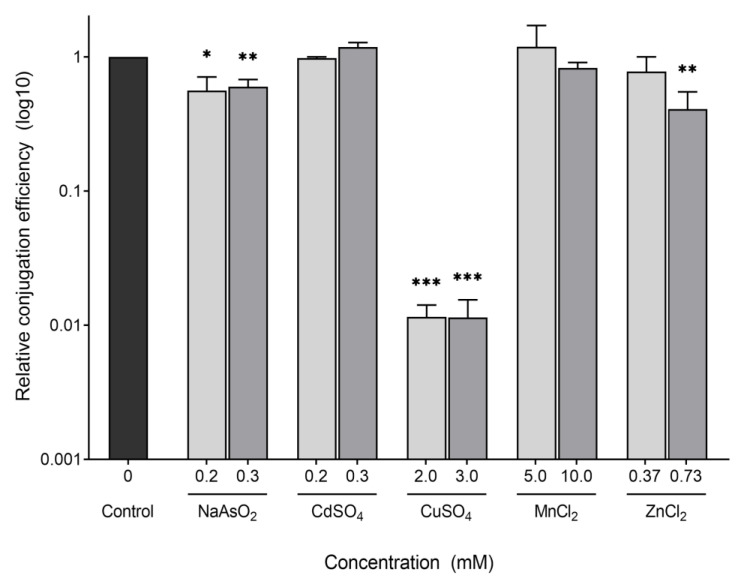
Effect of heavy metals on conjugation efficiency of an F-plasmid in *E. coli*. Donor and recipient strains were grown to log phase and then allowed to mate in the presence of heavy metals at the indicated concentrations. Conjugation efficiency is expressed as the ratio of transconjugants formed per donor. Bars represent the mean conjugation efficiency relative to the control (no heavy metal added), and the error bars are the standard error of the mean (SEM) of 2–6 biological replicates with 2 technical replicates each. *p*-values were computed using a one-sample unpaired *t*-test comparing the sample mean to a hypothetical value of 1 (the mean relative conjugation efficiency of the control). The level of significance is indicated as follows: * *p* < 0.05, ** *p* < 0.01, *** *p* < 0.001.

As shown in Figure 1, the most drastic effect on conjugation was for CuSO_4_ at 2 mM, with a decreased conjugation efficiency of almost 100-fold compared to the control that was not exposed to any chemical. A less drastic, but still significant, decrease was seen for 0.2 mM NaAsO_2,_ which showed an approximately 2-fold decrease; while the other chemicals caused no significant change. Since many of the chemicals showed little to no effect on the conjugation efficiency of our donor strain, we decided to test slightly increased concentrations, to see if a higher concentration is needed to observe an effect. Again, a similar effect was seen for 3 mM CuSO_4_ and 0.3 mM NaAsO_2,_ which exhibited 100-fold and 2-fold decreases in conjugation efficiency, respectively. ZnCl_2_ (0.73 mM) also showed a significant decrease in conjugation efficiency of approximately 2.5-fold, while CdSO_4_ and MnCl_2_ still showed no significant changes. 

**Figure 2:**
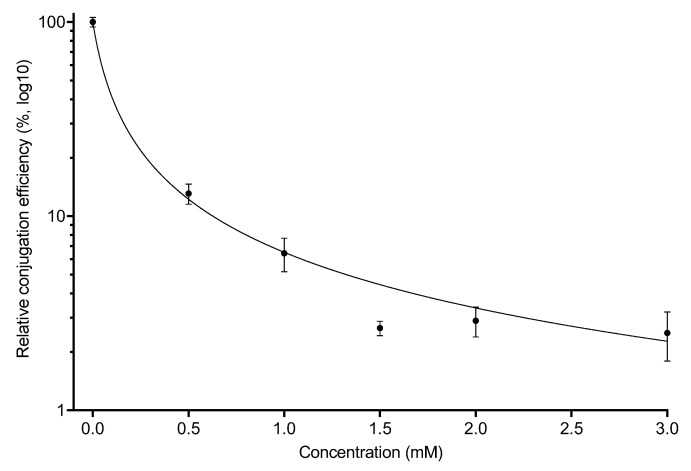
CuSO_4_ lowers conjugation efficiency proportional to the concentration added. Cultures of the donor (HA14) and recipient strain (HA4) were grown to log phase and then mixed and allowed to mate in the presence of various concentrations of CuSO_4_ for 30 min. Conjugation efficiency was calculated as in Figure 1 and as described in Materials and Methods. Symbols indicate the mean and error bars and represent the standard error of the mean (SEM) of 2 biological replicates with 2 technical replicates each.

Based on these results, we decided to follow up on our observations with copper. While some of the other chemicals showed a slight decrease in conjugation efficiency, copper displayed the most consistent and dramatic effect, and thus, we decided only to pursue CuSO_4_ to further investigate its effect on conjugation. We tested lower concentrations of CuSO_4_ and observed an effect proportional to the concentration, ranging from a decrease in conjugation efficiency of 10-fold at 0.5 mM to 100-fold at 3 mM (Figure 2).

### 2.3. The Effect of CuSO_4_ on Conjugation Is Not Due to Lower Expression from the P_Y_ Promoter

**Figure 3:**
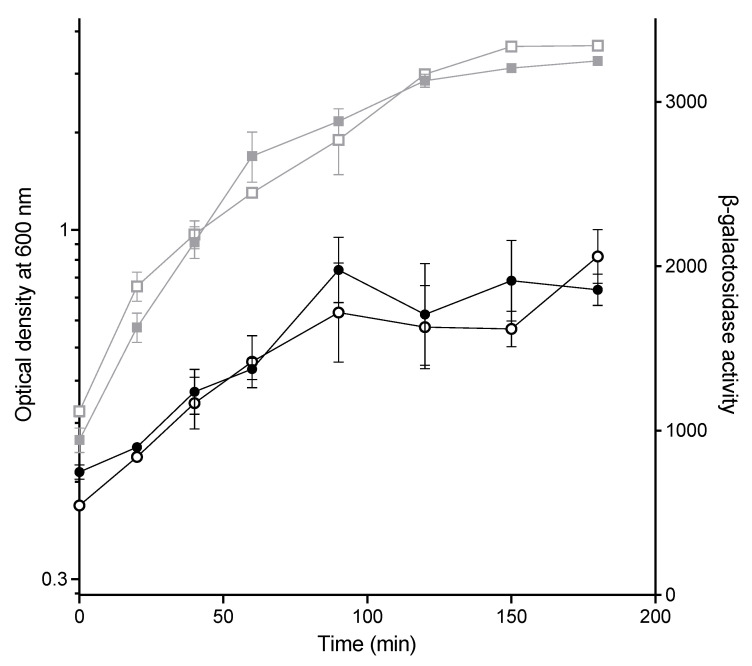
CuSO_4_ does not alter expression from the P_Y_ promoter. A P_Y_-*lacZ* strain (PAS3) was grown to log phase, after which the culture was split and 3 mM CuSO_4_ added (open symbols) or not added (closed symbols). OD600 is shown as gray squares and β-galactosidase activity as black circles. Symbols represent the mean, and the error bars are the standard error of the mean (SEM) of 2 biological replicates.

One way that CuSO_4_ could affect conjugation is by altering the expression of the machinery necessary for the transfer of the plasmid. For example, induction of stress responses in the donor could alter F-plasmid gene expression, as has been shown previously [47,48,49]. The conjugative machinery is primarily encoded by the major *tra* operon on the F-plasmid, which is under the control of the P_Y_ promoter. To investigate whether the exposure to CuSO_4_ affects the expression from the P_Y_ promoter, we constructed a P_Y_-*lacZ* strain (PAS3), let it grow to log phase, added 3 mM CuSO_4_, and measured the levels of β-galactosidase in the culture over time following exposure (Figure 3). No significant differences were observed between the cultures exposed to CuSO_4_ and those that were not. It should be noted that the effect on growth in this strain was somewhat variable, but where slight decreases in growth were seen, repetitions of this experiment also did not show any change in P_Y_-*lacZ* activity. These results indicate that the decrease in conjugation efficiency is not caused by changes in the expression of the major conjugative operon of the F-plasmid. 

### 2.4. CuSO_4_ Is Required during the Mating to Strongly Inhibit Conjugation

**Figure 4:**
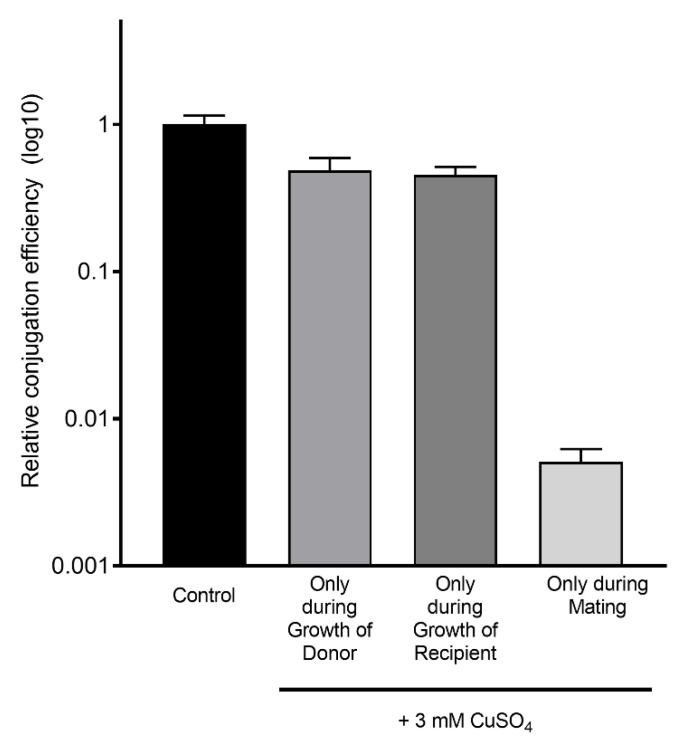
CuSO_4_ lowers conjugation efficiency when present during mating. ‘Donor’/’Recipient’: the donor (HA14) or recipient strain (HA4) was grown to log phase while exposed to 3 mM CuSO_4_, after which the culture was pelleted and resuspended in fresh LB, mixed with untreated donor or recipient, and allowed to mate in the absence of CuSO_4_. ‘Mating’: the donor and recipient strains were grown to log phase in the absence of CuSO_4_, and then allowed to mate in the presence of 3 mM CuSO_4_. The bars represent the mean conjugation efficiency relative to the control (0 mM), and the error bars represent the standard error of the mean (SEM) of 2 biological replicates with 2 technical replicates each.

Although *P_Y_*-dependent gene expression was unaltered, CuSO_4_ could affect conjugation by changing another aspect (e.g., assembly of pilus), and thus decrease conjugation efficiency. We reasoned that if we exposed the cells to copper during growth, followed by subsequent washing, any physiological change would be present for at least some of the time during the mating. In contrast, if copper was directly affecting the mating by another mechanism, copper would need to be present during the mating assay. Further, we wanted to know whether the effect was on the donor cell or the recipient cell. To test this, we exposed HA14 (donor) and HA4 (recipient) to 3 mM CuSO_4_ during growth to log phase, after which the cultures were pelleted and resuspended in fresh LB, mixed with the appropriate donor or recipient culture (unexposed to copper), and allowed to mate in the absence of CuSO_4_ (Figure 4). 

The matings where the donor or recipient were only exposed to 3 mM CuSO_4_ during growth, and not during the mating, showed only a 2-fold decrease in conjugation efficiency. In contrast, exposing the mating mixture to 3 mM CuSO_4_ decreased conjugation efficiency by approximately 100-fold compared to the control. Thus, we conclude that copper needs to be present during the mating for a substantial effect. We suspect that trace amounts of copper remaining in the mating mixture after the resuspension in fresh LB accounts for the 2-fold decrease seen.

## 3. Discussion

The question of whether heavy metals increase or decrease conjugation is important because ABR plasmids are spreading rapidly and bacteria are commonly exposed to heavy metals in the environment. For instance, copper surfaces (e.g., door handles) are being used in hospitals to promote continuous bacterial cell death and decrease the spread of pathogenic bacteria [50]. However, if in some cases, heavy metals can select for ABR and also increase conjugation, we may be creating more problems than we are solving.

Previous work has shown both increased and decreased conjugation in the presence of heavy metals [30,31,32,33,34,35], but it is difficult to compare the results from these papers. First, different Inc group plasmids were used, and we know that the genes/proteins involved in conjugation can vary, and may respond differently to stressors. Further, in the lab, cells carrying different types of plasmids require different amounts of time to effectively conjugate. This means that, in order to properly measure the conjugation efficiency, the mating mixture needs to be incubated for a longer time for some plasmids compared to others. This increased incubation time increases the impact that cell death [51] and co-selection [28] have on the results, especially when exposing the mating cells to potentially toxic compounds. Second, different strains, and even different species, are used in many of the papers cited below, and these could vary considerably in their response to a stressor. Finally, the ways in which conjugation rates are calculated varies greatly between papers. All of these factors make finding compounds that broadly affect conjugation difficult, and even comparing the effect of various compounds across different studies can be complicated. 

We investigated the effect of five heavy metals on the conjugation of an IncF plasmid in *Escherichia coli*. To minimize confounding factors, we chose a concentration of metals that had only a small effect on growth rate, and we only allowed the cells to mate for 30 min. Further, data were normalized to the number of viable donors at the end of the experiment, which minimized any differential effects of growth or death during the mating.

No significant effect on F-plasmid conjugation was observed with 0.2 and 0.3 mM CdSO_4_, or with 5 and 10 mM MnCl_2_. Previous studies have reported that exposing *P. putida* carrying RP4 (IncPα) to 0.055 and 0.55 mM CdCl_2_ increases conjugation rates. The authors found that this increase was caused by a reduction in the expression of the regulators *korA*, *korB,* and *trbA* [32]. However, these genes are not present on IncF plasmids, and this difference between IncF and IncPα plasmids clearly demonstrates how different plasmid groups (and species) are affected differently by stressors. In contrast, another group reported that adding 12.6 and 63.6 µM CdCl_2_ dramatically decreased the conjugation of pKJK5 (an IncP-1ɛ plasmid) from an *E. coli* donor [31], indicating that, in contrast to IncF, conjugation of this plasmid in *E. coli* was severely affected by cadmium. Note however, that in this experiment, in addition to the difference in plasmid type, matings were done under very different conditions with a mixture of bacterial recipients, making it difficult to compare with our experiments. No effect of manganese on conjugation has, to the best of our knowledge, been reported. 

NaAsO_2_ exhibited a statistically significant 2-fold decrease in conjugation efficiency at 0.2 and 0.3 mM. This result is similar to that reported for an IncP-1ɛ plasmid, where 40.5 and 125.2 µM decreased the conjugation efficiency by approximately 30 and 50%, respectively, compared to no stress [31]. Although these experiments were carried out under very different conditions, as noted above, this could indicate that the mechanism behind the change in conjugation efficiency is shared between the two Inc groups, or that it affects the cell in a more general way. Gualco et al. [39] showed that sodium arsenate increased recombinants in an Hfr mating, but it is difficult to separate mating efficiency from recombination in this paper.

The addition of 0.73 mM ZnCl_2_ decreased conjugation efficiency by 2.5-fold, but ZnCl_2_ had no significant effect at 0.37 mM. The difference in effect between the two concentrations indicates that the effect is concentration-dependent, and suggests that increasing the concentration further may decrease the conjugation efficiency even more. However, this will need to be balanced with the fact that our donor strain began to grow very poorly at 1 mM ZnCl_2_. Previous studies of Hfr matings (in a strain that was more resistant to zinc than our strain) showed that 1 mM ZnCl inhibited mating pair formation, an early step of conjugation [35], and this could be examined further. ZnCl_2_ has previously been reported to decrease the conjugation of an IncI1 plasmid by 58% at 0.37 mM and 90% at both 0.92 and 1.47 mM, as well as inhibiting the conjugation of an IncK plasmid by more than 98% at these concentrations [30], again highlighting differences in how the transfer of different plasmid families is affected by stress. 

Finally, CuSO_4_ had a drastic effect on the conjugation of the F-plasmid, with an approximately 100-fold decrease in efficiency at both 2 and 3 mM. Due to the extent of the effect of the addition of CuSO_4_ to the mating mixture, we performed some follow-up experiments. We found that the decrease in conjugation efficiency is dose-dependent, with a 10-fold decrease in efficiency observed at 0.5 mM, and that the effect observed with CuSO_4_ is not caused by changes in expression from P_Y_, the main promoter of the transfer operon of the F-plasmid. We also found that the decrease in conjugation efficiency is much less drastic if the donor or recipient cells are grown with CuSO_4_ and then the medium replaced with fresh medium, indicating that the effect is not primarily caused by changes in the physiology of the donor or recipient.

CuSO_4_ has previously been shown to decrease the conjugation of an IncK plasmid by reducing the expression of the plasmid-borne genes *nikB* and *traB* encoding a relaxase and a protein involved in the assembly of the T4SS, respectively [30]. However, our results show that the expression from the main operon of the F-plasmid, under the control of the P_Y_ promoter, was not impacted by the presence of CuSO_4_, indicating that the mechanism by which CuSO_4_ influences the conjugation of IncK and IncF plasmids is different. Zhang et al. [33,34] have shown that low concentrations of copper can increase conjugation using an RP4 (IncP⍺) plasmid system in *E. coli*. This was effective when the recipient was either *E. coli* or *P. putida*. However, Parra et al. [36] showed that copper sulfate and copper nanoparticles decreased conjugation of two different IncP plasmids in *P. putida*. This again highlights that different Inc groups and species, as well as other experimental design differences, can give very different results.

The exact mechanism of action of copper in killing bacteria is controversial [52]. Copper ions can be reduced and produce reactive oxygen species (ROS) in a Fenton reaction, and this has been presumed to be the major mechanism of copper-induced bacterial killing. However, other studies have suggested a direct interaction of copper or copper nanoparticles with the membrane of gram-negative bacteria, causing membrane disruption. Indeed, it has been shown that copper ions (Cu^2+^) can bind to phospholipids and reduce membrane fluidity [53]. Most likely, copper toxicity is a cascade effect, where membrane damage leads to a greater influx of copper ions, which then cause ROS-related damage as well as compete for heavy metal binding sites in enzymes [50]. 

We can thus speculate on the mechanism by which copper interferes with conjugation. The F-pilus is primarily composed of TraA pilin protein subunits, but it is also stoichiometrically associated with phospholipids: phosphatidylglycerol (PG) species PG 32:1 (16:0, 16:1) and PG 34:1 (16:0, 18:1) [54]. An increase in ROS could damage either the proteins or the phospholipids of the F-pilus, but we would not expect that to be rapidly reversible as was shown in this work. However, if copper reversibly binds to the phospholipids of the F-pilus, perhaps it prevents proper functioning of the pilus (e.g., retraction) when present in the mating mixture. We could use chelating and quenching agents [52] to test the relative contribution of copper ions and ROS on conjugation in future experiments.

In future work, we will test the most common and clinically important plasmids in a systematic way to enable us to make conclusions about the role of heavy metal exposure in the transmission of antibiotic resistance.

## 4. Materials and Methods

### 4.1. Strains, Culturing Conditions, and Chemicals

Strains of *Escherichia coli* used in this study are listed in Table 2. Cells were cultured in LB medium (10 g/L tryptone, 5 g/L yeast extract, 10 g/L NaCl) supplemented with carbenicillin (Car, 50µg/mL), chloramphenicol (Chl, 30 µg/mL), kanamycin (Kan, 50 µg/mL), streptomycin (Str, 100 µg/mL), and/or tetracycline (Tet, 10 µg/mL), where appropriate, at 37 °C unless specified otherwise. The chemicals and final concentrations tested in this study are listed in Table 1.

### 4.2. Growth Assay

Overnight cultures of the IncF donor, HA14, were diluted 1/100 in LB and grown to log phase (OD_600_ = 0.3–0.6). Upon reaching log phase, the culture was split into separate sub-cultures containing various concentrations of each of the chemicals tested (Table 1). The OD_600_ of the cultures was then measured every 30 min until the stationary phase was reached.

### 4.3. Liquid Mating Assay

Overnight cultures of HA4 (recipient) and HA14 (donor) were washed in prewarmed LB to remove the antibiotics used in the culture. The washed cultures were diluted 1/50 in prewarmed LB and grown to log phase (OD_600_ 0.3–0.6). Equal parts of the log phase donor and recipient cultures were mixed in a microcentrifuge tube containing the chemical to be tested, and then placed at 37 °C for 30 min to allow conjugation to occur. Control matings that were not exposed to any metal were included each day in every experiment. The matings were disrupted by vigorous vortexing for 1 min and then placing the cells on ice. Each mating mixture was serially diluted in 10-fold increments down to 10^−7^ using ice-cold 1 × M9 salts (3 g/L KH_2_PO_4_, 0.5 g/L NaCl, 6.7 g/L Na_2_HPO_4_, and 1 g/L NH_4_Cl). An aliquot of 10 µL of each dilution was spotted in duplicate onto LB plates containing Kan + Tet, and Chl + Tet, to select for donors and transconjugants, respectively. After quantification to measure the colony forming units/mL (cfu/mL) of the donors and transconjugants, the conjugation frequency was calculated as the number of transconjugants formed per donor. The mean conjugation efficiency in the matings exposed to metals was then normalized to that of the zero metal control mating that was performed on the same day, to account for day-to-day variation. Finally, the mean relative conjugation efficiency was calculated from the relative conjugation efficiencies of 2–6 biological replicates with 2 technical replicates each.

### 4.4. Construction of a P_Y_-lacZ Fusion Strain

A reporter strain for *tra* operon activity was constructed by replacing the *lac* promoter in the chromosome with the P_Y_ promoter of the F-plasmid. This construction was performed according to the system described by Fried et al. [55]. The P_Y_ promoter fragment from the F-plasmid of XL1-Blue (Stratagene) and flanking lac sequences from MG1655 were amplified by PCR (primers shown in Table S1), cloned, and then recombined into the chromosome of LF1 using Lambda Red recombineering [56]. Integration into the chromosome in PAS3 was verified by PCR using primers that bind outside of the constructed reporter, and subsequently sequenced.

### 4.5. β-Galactosidase Assay

The P_Y_-*lacZ* strain PAS3 was diluted 1/150 in LB and grown to log phase. Once in log phase, the culture was split into several smaller cultures, to which CuSO_4_ was added. Samples were taken to measure OD_600_ and β-galactosidase activity. The β-galactosidase activity was measured as previously described by Miller [57], with modifications [58], and activity expressed as (1000 × OD_420_)/(OD_600_ (cells) × sample volume (ml) × reaction time (minutes)).

### 4.6. Statistics

All statistical analyses were carried out using Prism GraphPad v. 9.3.1 (Dotmatics, Inc., Boston, MA, USA)

## Figures and Tables

**Table 1:** Chemicals tested in this study.

Compound	Chemical Formula	CAS Number	Initial Concentrations Tested (mM)	Concentrations Tested for Conjugation (mM)
Cadmium sulfate	CdSO_4_	10124-36-4	0.2, 0.3, 0.4, 0.5 and 0.6	0.2, 0.3
Copper sulfate	CuSO_4_	7758-98-7	0.5, 1.0, 1.5, 2.0 and 3.0	2.0, 3.0
Manganese chloride	MnCl_2_	13446-34-9	5.0, 10.0, 15.0, 20.0 and 30.0	5.0, 10.0
Sodium arsenite	NaAsO_2_	7784-46-5	0.2, 1.0, 2.0, 3.5 and 5.0	0.2, 0.3
Zinc chloride	ZnCl_2_	7646-85-7	0.37, 0.55, 0.73, 1.1 and 1.47	0.55, 0.73

**Table 2:** Strains used in this study.

Strain	Genotype	Resistance(s)	Source/Reference
HA4	BW25113 *araB*::Chl^R^	Chromosomal Chl^R^	[8]
HA14	BW25113 *argC*::Kan^R^ [F’ *proAB lacIq Z*∆*M15* Tn10]	Chromosomal Kan^R^Plasmid Tet^R^	[8]
LF1	MG1655 *rpsL150 P_lac_::rpsL-neo-kan::lacZ*∆^1–100bp^	Chromosomal Kan^R^	[55]
MG1655	F-λ-*ilvG-rfb-50rph-1*	-	Lab stock
PAS3	MG1655 *rpsL150 P_Y_-lacZ* [F′ *proAB lacIq Z*∆*M15* Tn10]	Chromosomal Str^R^Plasmid Tet^R^	This study
XL1-Blue	*recA1 endA1 gyrA96 thi-1 hsdR17 supE44 relA1 lac* [F′ *proAB* *lacI*^q^*Z*Δ*M15* Tn10]	Plasmid Tet^R^	Stratagene, Inc.

## Data Availability

Not applicable.

## Supplementary Materials

The following supporting information can be downloaded at: https://www.mdpi.com/article/10.3390/antibiotics11081123/s1, Figure S1: Growth curves of HA14 exposed to the heavy metals. Table S1: Primers used in this study
