# Supplementary material for: The Effect of Heavy Metals on Conjugation Efficiency of an F-Plasmid in Escherichia coli
Source: Antibiotics (Basel). 2022 Aug 19;11(8):1123. doi: 10.3390/antibiotics11081123 (PMC9404890; doi:10.3390/antibiotics11081123)
Supplement: Supplementary file 1 [file antibiotics-11-01123-s001.zip › antibiotics-1848908-supplementary.pdf]

Supplemental Materials

Palm, et al

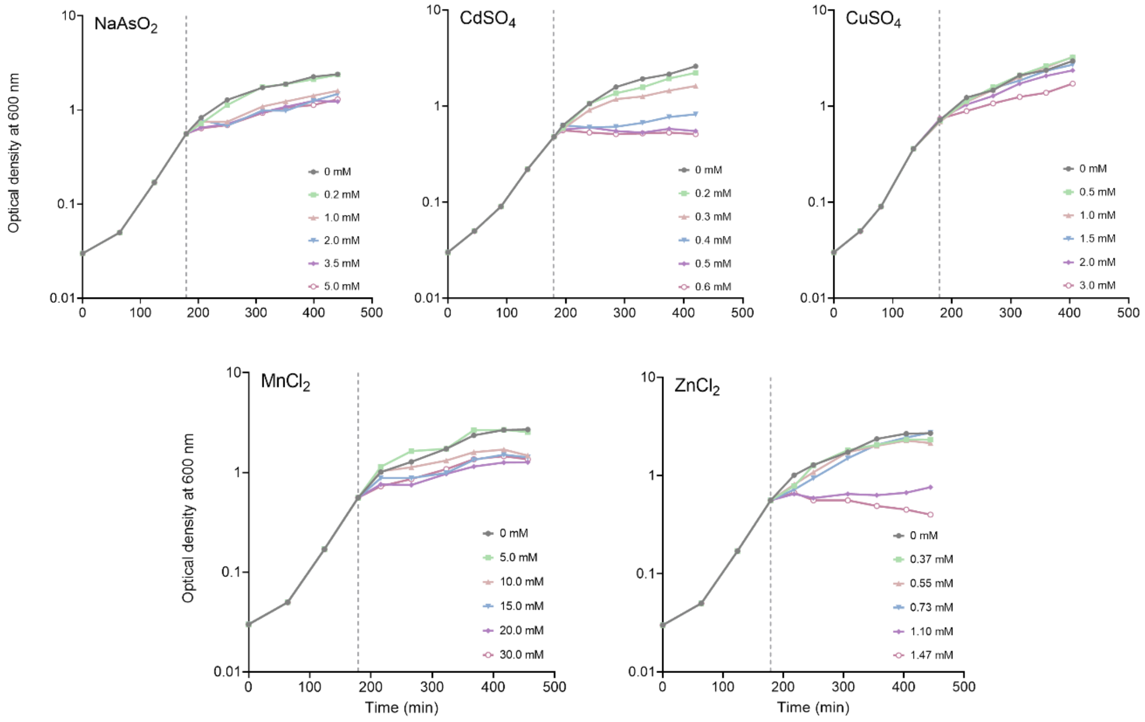

Figure S1. Growth curves of HA14 exposed to the heavy metals. HA14 was grown to log phase after which the culture was split into subcultures containing various concentrations of the chemicals tested. The OD_{600} of the cultures were measured until the cells entered stationary phase. The dashed line indicates when the culture was split and the chemical added.

Table S1. Primers used in this study

Primer name	Primer sequence (5’ -> 3’)
lacI.FOR	GAGCAGCATCACTGCCCGCTTTCCAG
lacI.REV	TTGGCCGATTCATTAATGCAGCTGGTCTGCGTCTGGCTGGCT
lacZ.FOR	CACAGACTTAGATTGGTATATATACGCATATGCGACTGTCCTGGCCGTAAC
lacZ.REV	GGGAGGTGTTAATGACCATGATTACGGATTCACTGG
PY.FOR	ATCATGGTCATTAACACCTCCCGCTGTTTATCT
PY.REV	GGGCAGTGATGCTGCTCATGTTCGTCA
